# Relationship between neutralizing and opsonizing monoclonal antibodies against foot-and-mouth disease virus

**DOI:** 10.3389/fvets.2022.1033276

**Published:** 2022-10-12

**Authors:** Artur Summerfield, Heidi Gerber, Rebeka Schmitt, Matthias Liniger, Santina Grazioli, Emiliana Brocchi

**Affiliations:** ^1^Institute of Virology and Immunology, Köniz, Switzerland; ^2^Department of Infectious Diseases and Pathobiology (DIP), Vetsuisse Faculty, University of Bern, Bern, Switzerland; ^3^Istituto Zooprofilattico Sperimentale della Lombardia e dell'Emilia-Romagna, Brescia, Italy

**Keywords:** FMDV, opsonization, antibodies, monoclonal antibody, Fc gamma receptors

## Abstract

Previous studies demonstrated that polyclonal antibodies against foot-and-mouth disease virus (FMDV) generated by vaccination can mediate immune functions not only through virus neutralization but also through promoting virus uptake by macrophages and dendritic cells that are otherwise resistant to FMDV infection. This causes abortive infections resulting in activation, enhanced antigen presentation but also cell death. Here we report the use of RAW264.7 cells representing a murine macrophage cells line to characterize opsonizing functions of a collection of monoclonal antibodies (mAbs) against FMDV O and A serotypes. We demonstrate that all neutralizing immunoglobulin G isotype mAbs are able to opsonize FMDV resulting in increased cell death of RAW264.7 cells. In contrast, neutralizing IgM antibodies did not possess this activity. Opsonization was observed with broader reactivity within the serotype when compared to neutralization. Importantly, the anti-O serotype D9 mAb reacting with the continuous epitope within the G-H loop of VP1 that contains the RGD binding site of FMDV, opsonized several FMDV serotypes despite its restricted neutralizing activity within the O serotype. Furthermore, by generating RAW264.7 cells expressing bovine CD32, an easy-to-use cell-based assay system to test for bovine antibody-dependent enhanced infection of FMDV was generated and tested with a collection of sera. The data indicate that opsonizing titers correlated better with vaccine dose when compared to neutralizing titers. On the other hand, neutralization and opsonization titers were similar predictive of protection. We conclude that low avidity interactions are sufficient to mediate Fcγ receptor-mediated immune functions that could contribute to protective immune responses against FMDV.

## Introduction

Foot-and-mouth disease virus (FMDV) is a member of the *Picornaviridae* family, and causes the high impact and very contagious foot-and-mouth disease (FMD) affecting cloven-hoofed animals. Although the disease is preventable by inactivated vaccines, proper vaccine selection is of crucial importance and needs to take into consideration not only seven known serotypes of FMD virus (FMDV; O, A, C, Asia-1, South African Territories 1, 2, and 3) but also the enormous antigenic variation within one serotype. This is caused by a high mutation rate of this RNA virus that strongly affects viral proteins targeted by neutralizing antibodies. Nevertheless, despite the central importance of neutralizing antibodies in protective immunity against FMDV, animals can be protected with low levels or in absence of neutralizing antibodies (1, 2). Previous studies demonstrated that polyclonal serum antibodies generated by vaccination can mediate immune functions not only through virus neutralization but also through promoting virus uptake by Fc gamma receptors (FcγR) expressed on macrophages and dendritic cells (DC) that are otherwise resistant to FMDV infection (3–5). In fact, opsonization of virus antibody complexes has been demonstrated to be a crucial component of the immune response in a mouse model, required for the final *in vivo* destruction of the virus by phagocytes (5–7). In the case of plasmacytoid dendritic cells (pDC), such sera can greatly enhance FMDV-induced interferon-α secretion and could therefore be associated with a direct antiviral effect as well as the potent adjuvant effect of activated pDC for adaptive immune responses. Interestingly, sera with such activities were broadly cross-reactive even across different serotypes of FMDV (3).

Considering the above and the fact that field conditions usually represent heterologous challenge situations, analyzing such opsonizing antibodies (mAbs) is of relevance to understand protective immune responses. Using monoclonal antibodies, we therefore performed the present study to understand the relationship between neutralization and opsonization as well as the degree of cross-reactivity of opsonizing mAbs. To this end, we established a RAW264.7 cells-line based assay for both murine mAbs and bovine antibodies. Our data demonstrate that even at the monoclonal level opsonizing antibodies can be highly cross-reactive even across serotypes. All opsonizing mAbs identified were neutralizing against homologous viruses, indicating that low affinity is sufficient to mediate opsonization but not neutralization of FMDV. Considering these results, we also generated RAW264.7 cells expressing bovine CD32, to test the relationship of opsonizing with neutralizing activities and with the outcome of vaccination.

## Materials and methods

### Viruses

The following viruses were used: O_1_/SWI/65 (O_1_ Lausanne), O/BUL/1/91, O/GRE/22/96, O/GRE/ 21/94, O/VIE/7/97, A/MCD/6/96, A/MAY/6/96, A/TUR/99, A_24_/Cruzeiro/55, A/SAU/17/92, and Asia-1/TUR/6/2000, C-S8cl (C_1_ SPA/7/79). Viruses were kindly provided by the World Reference Laboratory for Foot-and-Mouth Disease of The Pirbright Institute, UK), with the exception of S8cl representing a plaques-purified C_1_ virus kindly obtained from Francisco Sobrino (Centro de BiologMolecular Severo Ochoa, Madrid, Spain). Virus stocks were made using Baby Hamster Kidney (BHK) 21 cells that were grown in Glasgow's minimum essential medium (GMEM, Thermofisher, Gibco) supplemented with 5% v/v Fetal Bovine Serum (FBS, Biowest S05595S1810). Isolates of FMDV were propagated in BHK-21 cells and viral titres were determined by end-point titration on BHK-21 cells as previously described (8). Mock antigen was prepared from uninfected BHK-21 cells in the same manner as FMDV.

### Monoclonal antibodies

The present study employed mAbs against FMDV O_1_/SWI/65, O_1_/Manisa/TUR/69, A_24_/Cruzeiro/55 and A/MAY/6/96 (Table 1). All mAbs were generated at the Istituto Zooprofilattico Sperimentale della Lombardia e dell'Emilia-Romagna (IZSLER) using standard methods of mouse immunization, hybridoma technology and screening (14–16). The mAbs employed originated from mouse ascites fluids (generated over 20 years ago), except for mAb B2 which was from hybridoma cell culture supernatant.

**Table 1:** Monoclonal antibodies.

**mAb**	**Antigen**	**Isotype**	**Neutralization**	**Epitope**	**Source/reference**
1C12	O1/Manisa/TUR/69	IgM/IgG1	Yes	Site 2	IZSLER
1C6	O1/SWI/65	IgG2a	Yes	Site 2	IZSLER; (9, 10)
2A10	O1/Manisa/TUR/69	IgG1	Yes	Site 2&3	IZSLER
3C8	O1/SWI/65	IgM	Yes	Site 3	IZSLER; (9, 10)
B2	O1/SWI/65	IgG1	Yes	VP1, site 1	IZSLER; (9, 10)
D9	O1/SWI/65	IgG2a	Yes	VP1, site 1	IZSLER; (9, 10)
3B11	O1/Manisa/TUR/69	IgM/IgG1	No		IZSLER; (11)
A8	O1/SWI/65	IgG1	No	VP1	IZSLER; (10, 12)
4B10	A/MAY/6/96		Yes		IZSLER
4B12	A/MAY/6/96		Yes		IZSLER
4E9	A/MAY/6/96		Yes		IZSLER
4H2	A/MAY/6/96		Yes		IZSLER
4H8	A24/Cruzeiro/55		Yes		IZSLER
5G3	A/MAY/6/96	IgM	Yes		IZSLER
2C7	A/MAY/6/96		No		IZSLER
4E10	A24/Cruzeiro/55		No	VP1	IZSLER
5F7	A24/Cruzeiro/55		No		IZSLER; (13)

### Virus neutralization and opsonization assays

The virus neutralization assay was performed based on previously published protocols (17). Briefly, the different mAbs were incubated at different log2-fold dilutions with 100 TCID_50_ of the different viruses in a total of 100 μl for 1 h a 37°C. Then, the mixture was added to BHK-12 cell monolayers and scored daily for cytopathogenic effects for maximum 4 days.

For the opsonization assays we employed the murine macrophages cell line RAW264.7 (ATCC) cultured in DMEM (Thermofisher Gibco 32430), 10% heat-inactivated FBS. The cells were seeded in 48 well plates at 2 × 10^5^ cells/well in 400μl medium and cultured for 4 days of culture at 37°C, 5% CO_2_. To generate immune complexes, FMDV (multiplicity of infection 5 TCID_50_/cell) was mixed with the mAbs at three different concentrations (10, 1 and 0.1 μg/ml; virus without antibody as negative control), or with the cattle sera dilutions (1:10, 1:100, 1:1000) in a total volume of 250 μl and incubated for 30 min at room temperature. After removal of the culture medium from the RAW264.7 cells, the immune complexes, virus and mock controls were added to the cells (250 μl/well) for 1h incubation at 37°C, 5% CO_2_. Then, the cells were washed twice with 0.5 ml medium, and 400 μl of fresh medium per well was added, and the plates were incubated for 48 h at 37°C, 5% CO_2_. The cells were harvested as previously described (18), centrifuged at 250 g, 4°C, 5 min and resuspended in 100–200 μl CellWash^®^ (Becton Dickinson) at 4°C and analyzed by flow cytometry for propidium iodide (PI) incorporation to determine the percentages of dead cells. For each culture, the values obtained with mock-infected cells was subtracted from the FMDV-infected cells. The opsonization assay for the mAbs was validated for background reactions using a pool of mouse IgG1 and IgG2a of unknown specificity (MOPC-21 and MOPC-173, Sigma-Aldrich) at the same concentrations as the FMDV specific antibodies. Based on the results, a negative cut-off of 3% PI^+^ cells was defined. For the cattle sera, the PI^+^ values of the pre-immune sera at the corresponding dilutions were subtracted from the post-vaccination sera. Opsonizing and neutralizing titres of sera was calculated using the Reed and Muench formula (19).

### Trapping ELISA

MAb reactivities against the different FMDV isolates were evaluated by a trapping ELISA (20). Briefly, mAbs were incubated with pre-titrated concentrations of viruses (supernatant of infected cells) that had been trapped using a rabbit immune serum raised against different FMDV isolates. The reactivity of field isolates with each mAb, used at the double saturating concentration, was expressed as a percentage of the corresponding reaction with the parental strain, assumed to be 100 % (15).

### Cloning of bovine CD32, plasmid construction, and transfection

The nucleotide sequence of the open reading frame encoding bovine CD32 FcγR was obtained from NCBI NM_174539.2. RNA was extracted from bovine PBMCs using the Nucleo-Spin RNAII kit (Macherey-Nagel, Switzerland). Reverse transcription was done with SuperScriptIII reverse transcriptase (Life Technologies) followed by PCR amplification, gel-purification of the PCR product of expected size and ligation into the TOPO vector (Life Technology) according to manufacturer's protocol. Plasmids were amplified in XL-1 blue *E. coli*. After verification of the CD32 sequence, the gene was subcloned into the pEAK8_HIS vector (21) at the restriction sites EcoR1/Xbal using standard cloning techniques to generate the pEAK8_CD32 plasmid, which was amplified and purified using NucleoBond kits (Macherey-Nagel). For protein expression, HEK293 cells were transfected with MirusTrans IT293 transfection agent (Mirus, USA). At 48 h post transfection cells were stained with mouse-anti human CD32 (clone AT10, Biorad) followed by rabbit-anti-mouse immunoglobulin (Ig)-RPE conjugate (DAKO, Denmark) and detection was performed by flow cytometry (FACSCalibur, Becton Dickinson, Basel Switzerland).

The lentivirus expression system utilized was as previously described (22, 23) using plasmids obtained from the laboratory of Dr. Didier Trono (http://tronolab.epfl.ch/ Ecole Polytechnique Federale de Lausanne, Switzerland) through Addgene (Cambridge MA, USA). For subcloning of CD32 into the lentiviral transfer plasmid pWPT the pEAK8_CD32 plasmid was amplified by PCR using primers to insert the MIuI and Sall restriction sites present in the pWPT vector. The PCR product was digested using MIuI and Sall and the CD32 gene ligated into the pWPT vector using standard techniques. The plasmid was amplified in chemo-competent bacteria (Stbl2) and sequenced. HEK293 cells were transfected using the calcium phosphate precipitation method with the envelope plasmid (pMD2G), the packaging plasmid (pCMV-R8.74) and the transfer vector encoding bovine CD32 (pWPT _CD32). Medium was changed after overnight incubation at 37°C and the supernatant harvested after 48 h, centrifugated and filtered. The virus was purified and enriched by centrifugation on a 20% sucrose cushion at 28,000 *g* for 90 min, 4°C. For the generation of the RAW264.7 cell lines the cells were transduced twice with 1:100 dilution of the purified lentiviruses in 1 ml serum free medium of a T25 cell culture flask followed by culture overnight at 37°C and medium change between the transductions. Transduction efficiency was controlled by staining with anti-CD32 as described above and found to remain stable over at least three passages (76% positive, Supplementary Figure 1), during which the experiments were performed.

### Immunization of animals and source of serum samples

The sera used in the present study originated from vaccine trials performed in 2011. The first study was designed to test the protective capacity of the quadrivalent FMD vaccine Aftovaxpur^®^ T-1978A (O Middle East, Asia-1 Shamir, SAT2 Saudi Arabia, C Noville antigens; Merial, France; now Boehringer Ingelheim) to protect against FMDV O/BUL/2011 challenge. Following guidelines of the European Pharmacopeia, 6-month old cattle were vaccinated with four animals receiving the full dose (containing ≥3 protective doses 50 per valency), five animals ¼ dose and again five animals 1/20 dose. A second vaccination trial was performed with a vaccine (identical formulation as first trial) containing O3039, O_1_ Manisa or both O_1_ Manisa and O3039 antigens (full doses). For both studies, after 21 days a challenge infection with FMDV O/BUL/2011 (10,000 ID_50_) was done intra-dermo-lingually. Animals showing FMDV lesions were immediately euthanized and recorded as non-protected. Serum was collected before vaccination and weekly up to 7 weeks post vaccination.

### Ethics statement

The animal experiments were performed in compliance with the Swiss animal protection law (TSchG SR 455; TSchV SR 455.1; TVV SR 455.163). The procedures were reviewed by the committee on animal experiments of the canton of Bern, Switzerland, and approved by the cantonal veterinary authority (Amt für Landwirtschaft und Natur LANAT, Veterinärdienst VeD, Bern, Switzerland) under the license numbers BE95/10.

## Results

### Relationship between neutralization and opsonization by mAbs

**Figure 1:**
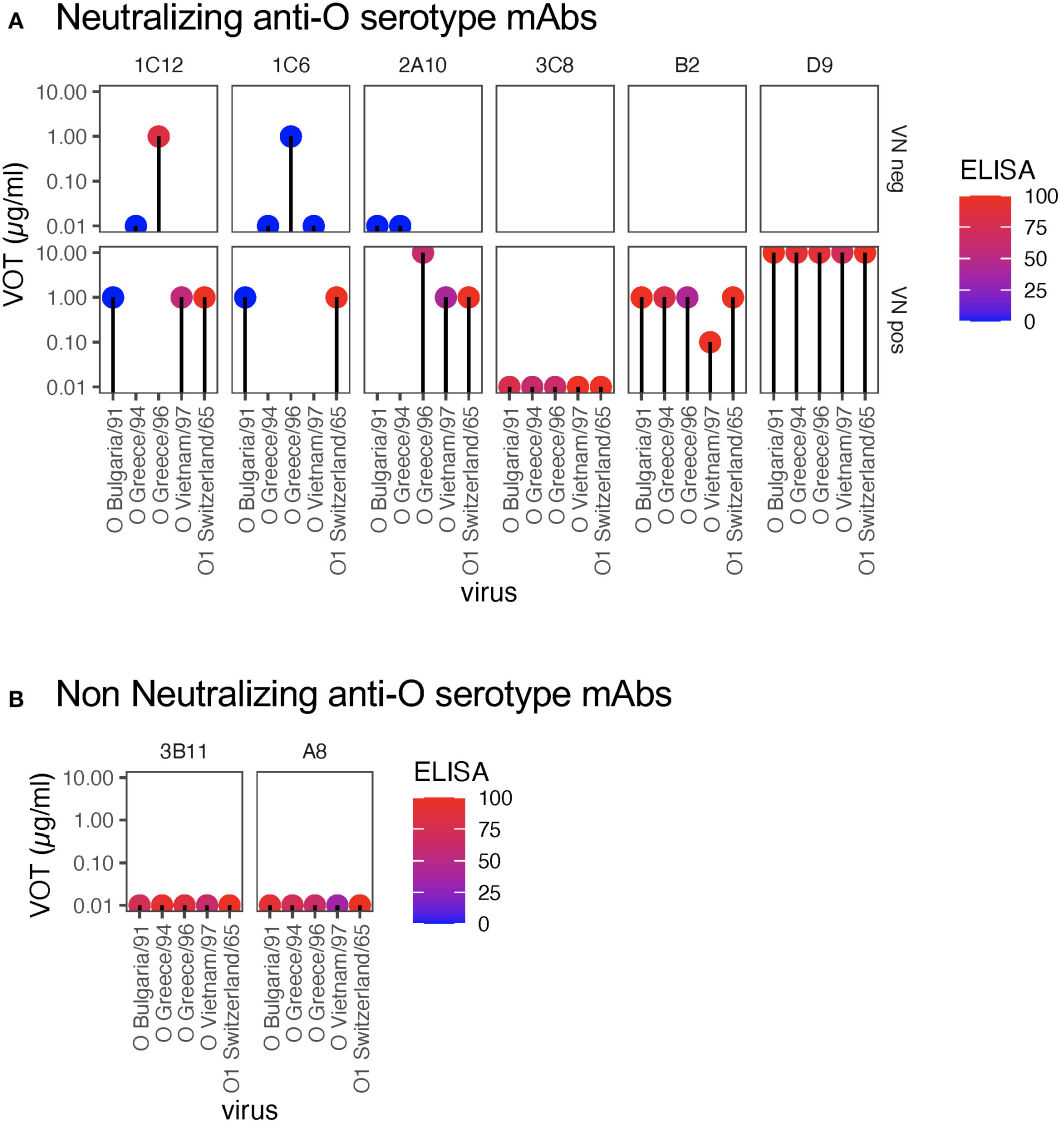
Virus opsonizing titers of mAbs against serotype O FMDV. VOTs were determined based on RAW264.7 cell death following incubation with mAbs (used at 10, 1, or 0.1 μg/ml) and virus (5 TCID_50_/cell). For each virus (x-axis), the virus opsonization titres (VOT) are shown by the y-axis levels of the dots. Each plot shows the data for a different mAb (all anti-O serotype, see Table 1). The colors of the dots reflect the ELISA reactivity as indicated in the legend. The panels in **(A)** show the data for mAbs that neutralize homologous virus. To visualize the relationship of VOT to neutralization, the data was separated by the ability of a specific mAb to neutralize the different O FMDV viruses. The upper panels show the VOT data for mAb-virus pairs without neutralization (“VN neg”), while the lower panels represent mAb-virus pairs with neutralization (“VN pos”). In **(B)**, the data for mAbs with no neutralizing activity against homologous virus are shown.

To determine the relationship between neutralization and opsonization, we tested the opsonizing activity of six anti-O serotype mAbs generated toward O_1_/SWI/65 or O_1/_Manisa/TUR/69 against a collection of O serotype FMDV isolates. Figure 1A shows the opsonizing activities of mAbs with neutralizing activity (at least against homologous FMDV, see Table 1). All neutralizing anti-O serotype mAbs with neutralizing activity against a particular FMDV isolate also possessed opsonizing activity, except for the IgM mAb 3C8. MAbs 1C12, 1C6, and 2A10 were unable to neutralize certain FMDV O isolates (row labeled “VN neg”). Nevertheless, in two cases these mAbs still had opsonizing activity (e.g., 1C12 and 1C6 opsonized O/GRE/96 in absence of neutralization). In one case we also had opsonizing activity for antibodies that lacked ELISA reactivity to that FMDV isolate (1C6, blue dot).

Figure 1B shows the data for two mAbs which are highly reactive in ELISA but non-neutralizing (including homologous virus): both also lacked opsonizing activity.

**Figure 2:**
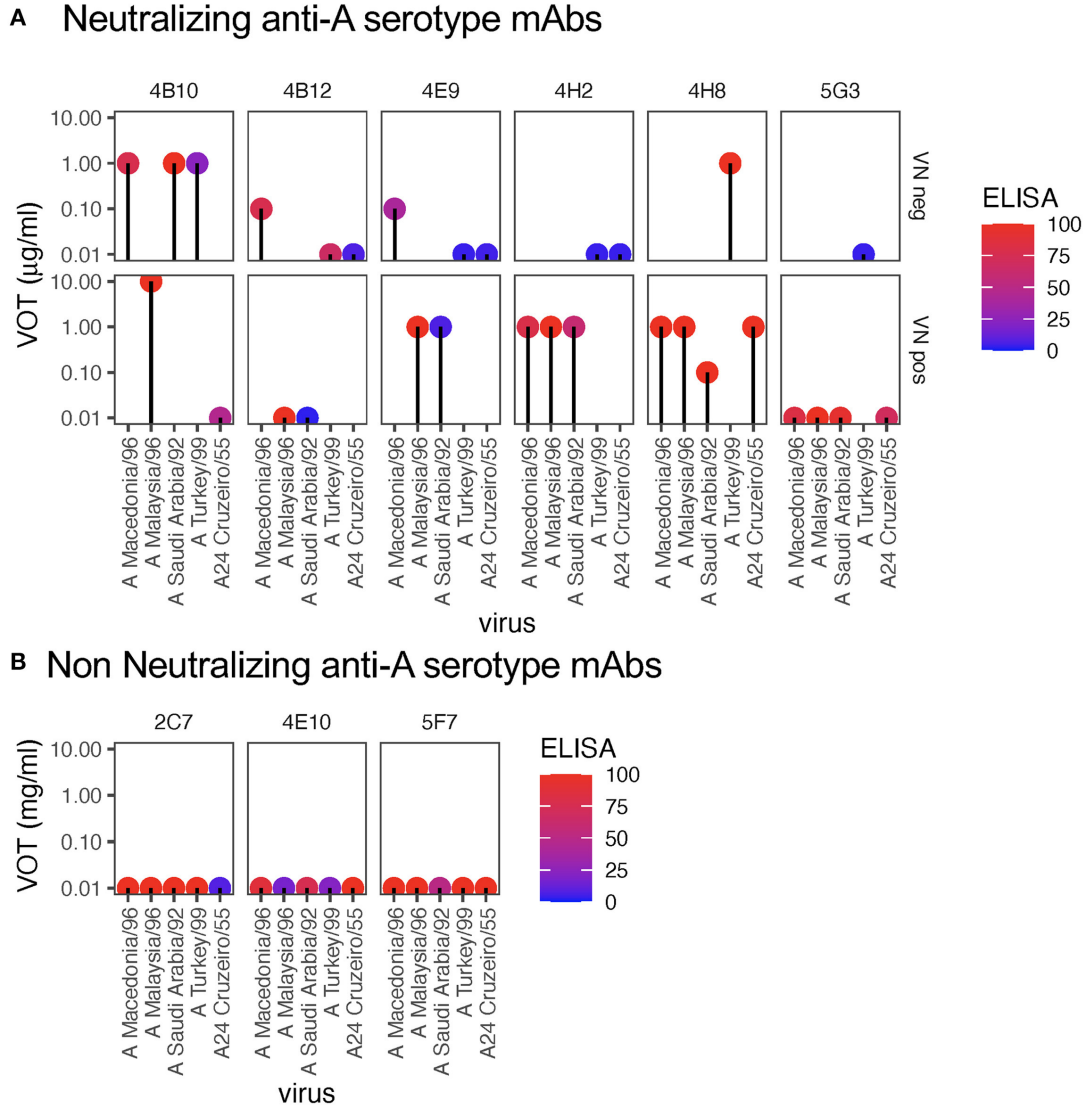
Virus opsonizing titers of mAbs against serotype A FMDV. For each virus (x-axis), the dots show the virus opsonization titers (VOT, y-axis), with the panels representing different anti-A serotype mAbs (see Table 1) as in Figure 1. The colors of the dots reflect the ELISA reactivity (legend). The panels in **(A)** show the data for mAbs that neutralize homologous virus. The upper panels show the VOT data for mAb-virus pairs without neutralization (“VN neg”), while the lower panels represent mAb-virus pairs with neutralization (“VN pos”). In **(B)**, the data for mAbs with no neutralizing activity against homologous virus are shown.

Considering the above results, we also tested a collection of mAbs generated against A/MAY/6/96 or A_24_/Cruzeiro/55 for activities against a collection of FMDV A isolates. Again, as visible in Figure 2A, the mAbs with neutralizing activity 4B10, 4E9, 4H2, and 4H8 also possessed the ability to opsonize FMDV. Nevertheless, although 5G3 was neutralizing it did not opsonize FMDV. Like 3C8 (Figure 1A), 5G3 is an IgM isotype. The upper row of Figure 2A confirms that for four of the mAbs opsonizing activity against certain FMDV isolates was found in absence of neutralization. This confirmed the opsonization was broader in its activity with antigenically divergent isolates within a serotype as previously reported using sera (3).

Figure 2B shows that non-neutralizing anti-A serotype mAbs were not able to opsonize despite high activity in the ELISA.

Taking together, the data indicate that at the monoclonal level only or mainly neutralizing IgG antibodies can opsonize FMDV. Furthermore, opsonization reactivity for many mAbs is broader than neutralization, and occasionally reactivity is even found in absence of ELISA positivity.

### Broad opsonizing activity of mAb D9 for A and O FMDV serotypes

**Figure 3:**
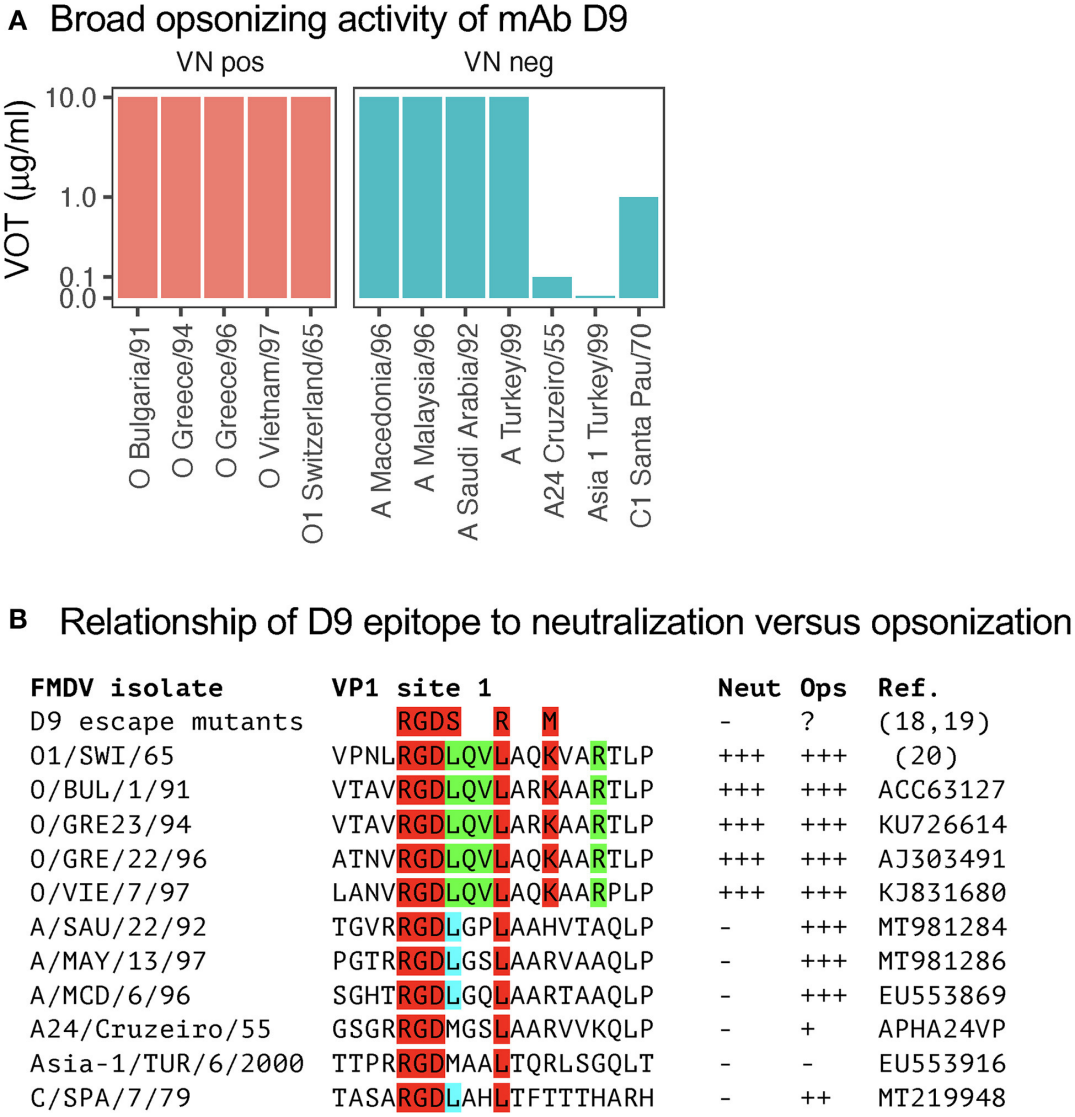
Virus opsonizing titers of mAb D9 against heterologous serotypes and its epitope relationship. In **(A)**, VOTs were determined as for Figure 1 but included viruses from the A, Asia-1, and C serotypes of FMDV. The data was separated by the ability of a specific mAb to neutralize the different FMDV viruses tested in neutralization negative mAb-virus pairs (right panels in blue, “VN neg”) and neutralization positive mAb-virus pairs (left panels in red, “VN pos”). In **(B)**, the amino acid sequences of the D9 epitope from the viruses used in **(A)** are shown together with their neutralizing and opsonizing activities. The amino acids depicted in red and green are common to all viruses neutralized by D9. The leucine depicted in blue is found in all viruses that are well opsonized by D9.

Considering the broad reactivity of mAb D9 within the tested O serotype viruses for both neutralization and opsonization, we also tested FMDV belonging to other serotypes. Strikingly this antibody that was generated against O_1_/SWI/65 was highly opsonizing for four of the five tested FMDV A viruses and even opsonized a FMDV C virus (Figure 3A). Nevertheless, as expected D9 was not able to neutralize non-O FMDV (Figure 3B).

The D9 represents one of the mAb generated in the early days of hybridoma technologies against O_1_/SWI/65, and its binding site was established to be a linear epitope near the integrin binding site of FMDV (24). By sequencing of D9 escape mutants and using synthetic peptides, the minimal epitope for binding was found be amino acid position 141–154 of VP1. More precisely, D9-escape mutants retain the conserved RGD motif (position 141–143) but had mutations of the leucines on position 144/147 and of the lysin on 150 [Figure 3B; (9, 24, 25)]. Also, our data confirmed that O FMDV isolates that were neutralized by D9 were conserved for the RGDLQVL–K–R amino acid stretch. In contrast, the common motif for opsonization only was found to be RGDL–L (9, 24). These results demonstrate that opsonization of FMDV by antibodies requires less stringent binding, which explains their relatively broad reactivity even across different serotypes.

### Relationship of opsonizing and neutralizing activity in cattle sera

We next employed the same methodology to sera from vaccinated cattle with the aim to understand the relationship between neutralization and opsonization with polyclonal sera in a relevant biological context. To this end we generated RAW264.7 expressing bovine CD32 to ensure efficient interaction of bovine immune complexes with the murine macrophage cell line.

**Figure 4:**
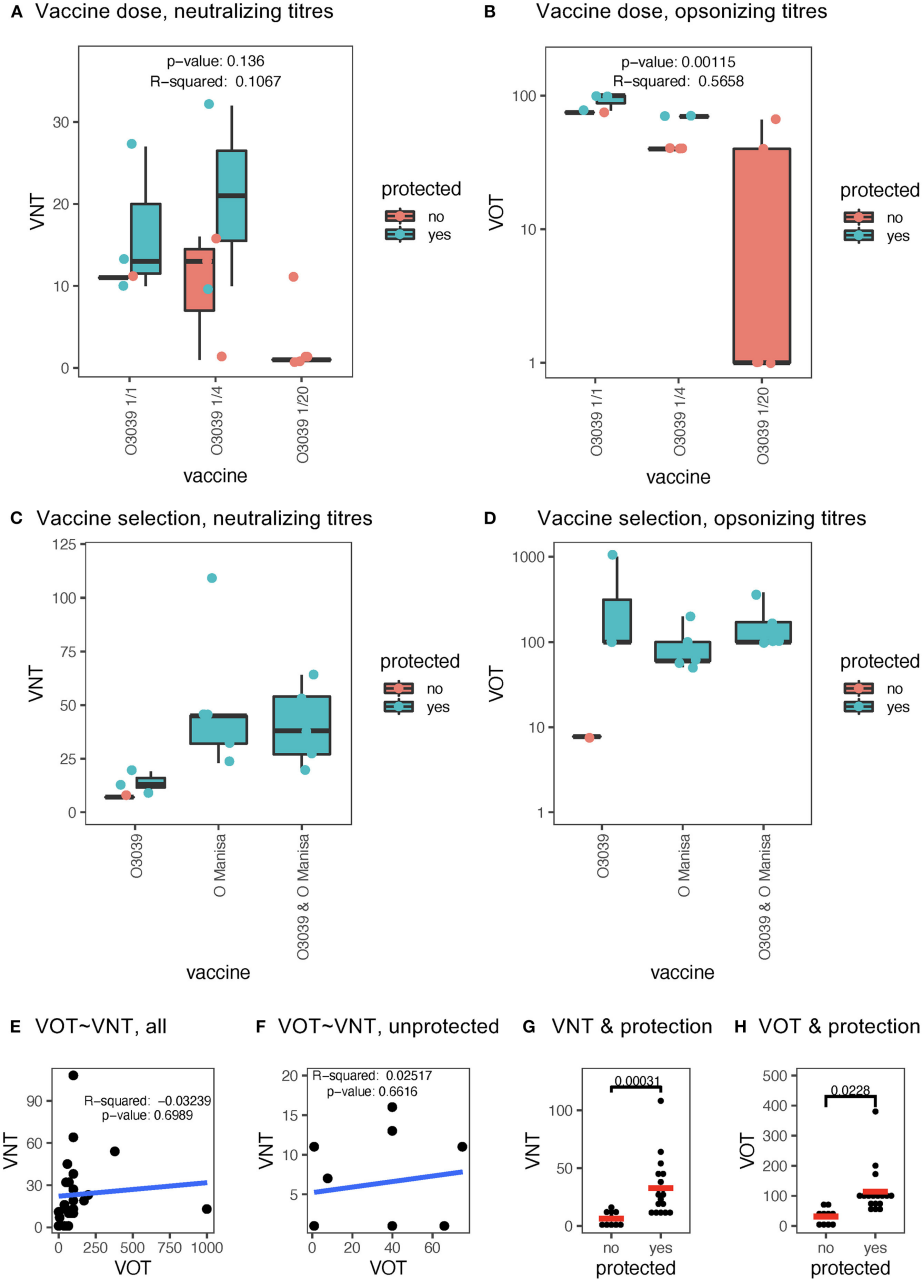
Relationship of VNT and VOT to vaccine dose, vaccine matching and protection. **(A)** Shows a boxplot of the VNT of cattle sera collected 21 days post vaccination using a multivalent FMD vaccine (T-1978) at full (:1), quarter (:4), and 1:20 dose (:20). In **(B)**, the same sera were analyzed for VOT. For the VOT's the propidium iodide positive values obtained with pre-immune sera at each serum dilution and from the same animal was subtracted from the sera obtained after vaccination. The *p* and *r*^2^ values in **(A,B)** originate from a linear regression analyses of the vaccine dose vs. the antibody tests. In **(C,D)**, boxplots of the VNT and VOT, respectively, induced by three different vaccine antigens (x-axis) are shown. Statistical significance was calculated with the Kruskal-Wallis test with corrections for multiple comparisons using the Benjamini and Hochberg test. For the boxplots in **(A–D)** the black horizontal bar shows the median, with the lower and upper hinges of the box plot indicating the 25 and 75th percentiles. The ends of the whiskers show the maximum and minimum values being at most 1.5 times the inter-quartile range the of the hinge. Dots represent values of individual animal (turquoise = protected; red = non-protected). **(E,F)** represent dot plots for the VOT vs. VNT tests for all data points and only for non-protected cattle, respectively (*r*^2^ and *p*-values from Spearman correlation analyses). In **(G,H)**, shows dot plots comparing protected and non-protected animals for their VNT and VOT, respectively. The *p*-values were determined by a Mann-Whitney *t*-test.

The first set of sera employed were from a vaccination-challenge experiment in which the multivalent vaccine Aftovaxpur^®^ T-1978A (Merial), was tested in three different doses in cattle following European Pharmacopeia guidelines. Only three out of four animals with the full dose, two out of four with the quarter dose and none of the 1/20 dose were protected against O/BUL/2011 challenge. In general, all vaccinated animals had only low or no neutralizing activity. Nevertheless, while none of the protected animals lacked neutralizing activity, three of the seven non-protected animals had VNT's at levels comparable to protected animals (Figure 4A). This contrasted with the VOT that reached levels that were ~10x higher and correlated with the vaccine dose. However, also in this test the VOT levels did not separate protected from unprotected animals (Figure 4B). In this scenario of insufficient matching of vaccine antigen with challenge virus, a linear regression analyses indicated that vaccine dose would impact the VOT, but not the VNT (Figures 4A,B).

We also applied the same tests to sera from a second vaccination/challenge experiment performed in cattle, that was as a follow-up to the first challenge experiment with the aim to identify matching vaccine antigens against O/BUL/2011. In this experiment, all animals except one were protected, and animals receiving a vaccine containing the O Manisa FMDV antigen were all protected. These animals induced higher levels of neutralizing antibodies as compared to the O3039 antigen (Figure 4C). In contrast, the levels of VOT were similar with all three FMDV antigens. Interestingly, the only non-protected animal had lower levels of VOT as compared to the protected cattle (Figure 4D).

Considering these results, we performed further statistical evaluations of the cattle data. As expected, there was no correlation between VNT and VOT, when all data were used or only the data from the non-protected cattle (Figures 4E,F, respectively). Nevertheless, when all data available were pooled, significantly higher levels of both VNT and VOT were found in protected compared to non-protected animals (Figures 4G,H).

Taken together, the cattle data indicate that VOT represent a sensitive measurement for vaccine-induced antibodies that correlate with vaccine antigen dosing but does not appear to be superior compared to VNT measurements in prediction protection.

## Discussion

Virus neutralization mediated by antibodies represents an *in vitro* measured functional phenotype mediated by high affinity interactions between the antigen binding (Fab) region of the antibody and a viral structure that typically prevents infection of cells and viral spreading within a cell culture. While neutralization assays are most useful and important functional assays often related to the protective value of antibodies, they are unable to measure antibody functions mediated by the Fc region of the antibody molecules through binding to FcR and complement. By binding to FcγR, IgG-antigen complexes can mediate endocytosis or phagocytosis followed by destruction of immune complexes, as well as activation of innate immune functions in Fc receptor bearing cells. These are typically myeloid cells, such as neutrophils, monocytes and macrophages, DC and natural killer cells (26). For FMDV, FcγR-mediated antibody functions related to macrophage or DC activation were previously demonstrated (3–7). In mouse models, these functions have been shown to be important in protection against FMDV (5, 6) and other virus infections (27).

For one of our aims, which was understanding the relationship of neutralization and opsonization of mAbs, we selected the murine RAW264.7 based on their resistance of FMDV infection and the fact that they had been employed for FcγR based functions (28). Of note, in these cells FMDV antibody complexes promoted cell death, as previously observed with bovine monocyte-derived DC (4). With the bovine DC model, antibodies mediated FMDV infection of these otherwise virus-resistant cells, resulting in cell death. In the present study we did not further investigate the mode of RAW264.7 cell death, as we used the cell line solely as a tool to measure opsonizing antibody.

The data with the mAbs permitted two main observations. First, only neutralizing IgG antibodies were identified as able to opsonize FMDV. Second, opsonization reactivity with FMDV strains of varying antigenicity was often broader than neutralization. The anti-O serotype mAb D9 was even found to cross-react with serotype A FMDV isolates.

A possible explanation for these observations is based on the very small size of FMDV of 20 nm. Taking the distance between the two F(ab) fragments of an antibody molecule of 10 nm into consideration, half of the diameter of the virion is covered by only one antibody molecule. Consequently, antibodies binding any structure to the outside surface of the virion with sufficient affinity (or concentration) will sterically inhibit the interaction of FMDV with its receptor on the target cell. It can therefore be argued that antibody affinity, and not *per se* epitope targeting, should represent the main determinant of neutralization strength. Now, while neutralization will require a binding competition between the virus-receptor and the antibody-virus interactions, this competition is absent during opsonization, which is mediated by Fc-FcγR interactions. In addition, myeloid cells and DC expressing FcγR lack FMDV receptors. Therefore, opsonization of FMDV occurs both with high and low affinity antibodies, providing they can bind the surface of the capsid. In contrast, antibodies against the internal side of the capsid or against non-structural proteins of FMDV obviously cannot opsonize intact virions for structural reasons.

Based on these simple and fundamental structural facts, it is understandable that several reports are available that have employed affinity/avidity measurements of sera to predict the protective values following vaccination (29–33). Nevertheless, as explained above while such tests are suitable for high-throughputs testing, they do not quantify other important functions of antibodies, in particular opsonization. For these reasons, we also tested a collection of bovine sera from vaccination-challenge trials in the RAW264.7 cell-based assay. The results obtained confirmed the work with monoclonal antibodies in the sense that opsonization was still observed with higher serum dilution and even with a vaccine antigen badly matched with the challenge virus. This was in accordance with previous observations demonstrating a high level of cross-reactivity with porcine sera in a DC-based opsonization test (3). In the present study, we found that the correlation of opsonizing antibody titer to vaccine dose was superior than that of neutralizing antibodies. For predicting protection both tests appeared to provide useful but not absolute information. Nevertheless, the number of animals analyzed was too low to permit a final conclusion or even define cutoffs. Future studies are required to address the question is a combination of the two tests could improve vaccine matching and vaccine quality assessment.

In conclusion, the present study demonstrates that low affinity interactions are sufficient to mediate Fcγ receptor-mediated immune functions that could play a role in protective immune responses against FMDV. This novel test developed provides the bases to collect more data to determine the value of such antibodies as correlate of protection following vaccine-induced immune responses against FMDV.

## Data availability statement

The raw data supporting the conclusions of this article will be made available by the authors, without undue reservation.

## Ethics statement

The animal experiments were performed in compliance with the Swiss animal protection law (TSchG SR 455; TSchV SR 455.1; TVV SR 455.163). The procedures were reviewed by the committee on animal experiments of the canton of Bern, Switzerland and approved by the cantonal veterinary authority (Amt für Landwirtschaft und Natur LANAT, Veterinärdienst VeD, Bern, Switzerland) under the license numbers BE95/10.

## Author contributions

AS designed the study and wrote the manuscript with corrections from EB. HG, RS, ML, SG, and EB performed laboratory work or contributed essential reagents and results. All authors contributed to the article and approved the submitted version.

## Funding

This present work was in part supported by the European Union FP7 project FMD-Disconvac (grant agreement 226556), and by the Federal Office for Food Safety and Veterinary Affairs (BVET grant 1.10.12).

## Conflict of interest

The authors declare that the research was conducted in the absence of any commercial or financial relationships that could be construed as a potential conflict of interest.

## Publisher's note

All claims expressed in this article are solely those of the authors and do not necessarily represent those of their affiliated organizations, or those of the publisher, the editors and the reviewers. Any product that may be evaluated in this article, or claim that may be made by its manufacturer, is not guaranteed or endorsed by the publisher.
