# Supplementary material for: Relationship between neutralizing and opsonizing monoclonal antibodies against foot-and-mouth disease virus
Source: Front Vet Sci. 2022 Oct 12;9:1033276. doi: 10.3389/fvets.2022.1033276 (PMC9597200; doi:10.3389/fvets.2022.1033276)
Supplement: Supplementary file 1 [file Data_Sheet_1.docx]

Supplementary file for

Relationship between neutralizing and opsonizing monoclonal antibodies against foot-and-mouth disease virus

By Artur Summerfield, Heidi Gerber, Rebeka Schmitt, Matthias Liniger, Santina Grazioli, Emiliana Brocchi.


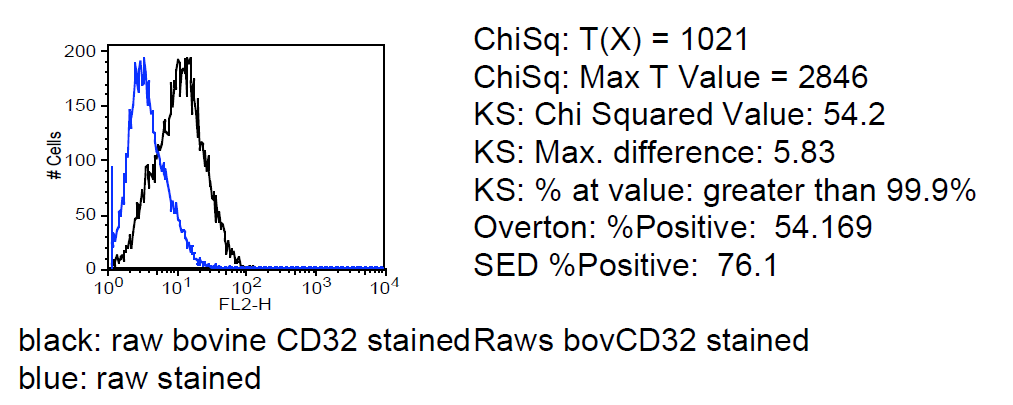


**Supplementary Figure 1.** Expression of bovine CD32 in murine RAW264.7 cells following transduction with bovine FCGR2A (CD32). RAW264.7 cells were transduced with lentiviruses encoding bovine CD32 in 1ml serum free medium of a T25 cell culture flask followed by culture overnight at 37°C, and a repeated lentivirus transduction. The cells were expanded including two passages and then staining with anti-CD32 (clone AT-10). The overlay histogram statistics calculated in the FlowJo software are shown.
